# The Role of Adjunctive Triamcinolone Acetonide in Post-traumatic Vitreoretinal Surgery: Current Insights and Future Perspectives

**DOI:** 10.7759/cureus.71040

**Published:** 2024-10-07

**Authors:** Anand Gandhi, Sachin Daigavane

**Affiliations:** 1 Ophthalmology, Jawaharlal Nehru Medical College, Datta Meghe Institute of Higher Education and Research, Wardha, IND

**Keywords:** anti-inflammatory, post-traumatic, proliferative vitreoretinopathy, triamcinolone acetonide, vitrectomy, vitreoretinal surgery

## Abstract

Post-traumatic vitreoretinal surgery is pivotal for repairing damage to the retina and vitreous body, commonly resulting from blunt or penetrating ocular trauma. Incorporating adjunctive pharmacological agents, particularly triamcinolone acetonide (TA), has gained considerable prominence in optimizing surgical outcomes. TA, a potent corticosteroid, is applied intraoperatively to improve the visualization of vitreous structures and epiretinal membranes, enabling surgeons to perform more precise maneuvers. Furthermore, its anti-inflammatory properties are instrumental in reducing postoperative complications such as macular edema and proliferative vitreoretinopathy (PVR), conditions that could hinder recovery and compromise visual acuity. TA use during vitrectomy enhances surgical precision and contributes to a smoother postoperative recovery. However, concerns surrounding potential side effects, such as steroid-induced ocular hypertension and cataract formation, necessitate careful patient selection and close monitoring throughout its use. Looking ahead, innovations in sustained-release formulations and combination therapies may further augment TA efficacy while reducing associated risks. This review provides current insights into TA application in post-traumatic vitreoretinal surgeries and highlights emerging trends poised to enhance its therapeutic role.

## Introduction and background

Post-traumatic vitreoretinal surgery involves the management of injuries that affect the retina and the vitreous body of the eye, often resulting in significant visual impairment if not addressed promptly [1]. These injuries can range from minor disturbances, such as vitreous hemorrhages, to more severe cases, such as retinal detachment or macular damage. Given the retina's delicate structure and essential function, surgical intervention plays a crucial role in preserving or restoring visual acuity [2]. The management of vitreoretinal trauma is complex, requiring specialized surgical techniques to address the injury and mitigate complications that may arise due to the trauma [2]. Vitreoretinal injuries are often caused by blunt or penetrating trauma, typically from incidents such as vehicular accidents, sports-related injuries, or physical assaults. Penetrating injuries usually occur due to high-velocity objects, such as metal fragments or glass, while blunt trauma may lead to internal compression or structural damage to the eye [3]. Retinal tears, vitreous hemorrhages, and retinal detachments are among the common consequences of such trauma, each posing its unique surgical challenges. Surgical repair aims to restore the anatomical integrity of the retina and vitreous body, minimize inflammation, and maximize functional recovery, particularly visual acuity [4].

The surgical management of post-traumatic vitreoretinal injuries has evolved considerably over the years. Modern techniques, such as pars plana vitrectomy, allow surgeons to access and repair the retinal structures, clear vitreous hemorrhage, and address retinal tear or detachment [5]. Depending on the complexity of the trauma, adjunctive procedures, including scleral buckling or intraocular tamponades (such as silicone oil or gas), may be necessary. These techniques are tailored to individual cases, with the primary objective of preventing complications such as proliferative vitreoretinopathy (PVR), which can lead to recurrent retinal detachment and further vision loss [5]. Adjunctive pharmacological therapies are increasingly being utilized to improve surgical outcomes in vitreoretinal trauma. Anti-inflammatory agents, corticosteroids, and antifibrotic drugs play a critical role in reducing postoperative complications, such as excessive inflammation and scarring [6]. These agents are essential in preventing secondary complications that could compromise the success of the surgery. By controlling inflammation and minimizing fibrosis, pharmacological adjuncts help ensure that the retina heals correctly and that long-term vision is preserved [6].

One such pharmacological agent, triamcinolone acetonide (TA), has emerged as a valuable adjunct in vitreoretinal surgery. TA is a corticosteroid with potent anti-inflammatory properties, widely used to manage ocular inflammation and enhance visualization during surgery. During vitrectomy, TA is injected into the vitreous cavity to stain the transparent vitreous and epiretinal membranes, enabling surgeons to visualize and remove these structures more effectively [7]. Moreover, TA helps reduce postoperative edema and inflammation, contributing to faster recovery and improved visual outcomes. Its application in post-traumatic vitreoretinal surgery has gained attention due to its ability to reduce intraoperative challenges and improve patient prognosis [7]. This review aims to explore the role of TA in post-traumatic vitreoretinal surgery, drawing from current clinical evidence and insights. By examining its effectiveness as an adjunct in improving surgical outcomes, reducing complications, and enhancing visualization, the review aims to provide a comprehensive understanding of its clinical utility. Additionally, future perspectives on the evolving role of TA in vitreoretinal trauma management will be discussed, highlighting innovations and emerging trends that may further optimize its use in surgical settings.

## Review

Triamcinolone acetonide: Mechanism of action

TA is a potent corticosteroid widely recognized for its versatile role in ophthalmic surgery, especially in managing post-traumatic conditions and vitreoretinal procedures. Its primary mechanism of action is rooted in its anti-inflammatory properties, which are crucial for reducing inflammation and promoting healing [8]. TA inhibits phospholipase A2, preventing cell membranes' breakdown and arachidonic acid release. This inhibition reduces the synthesis of pro-inflammatory mediators such as prostaglandins and leukotrienes. Furthermore, TA acts on nuclear factor kappa-B (NF-κB), suppressing the production of inflammatory cytokines such as interleukin-6 and interleukin-8. These combined actions make TA highly effective in managing ocular inflammation [9]. One of the key clinical benefits of TA is its ability to reduce macular edema, particularly in conditions such as uveitis. By stabilizing the blood-retinal barrier, TA minimizes fluid accumulation in the retina, significantly improving visual outcomes [10]. Clinical studies have demonstrated that intravitreal injections of TA can be as effective as anti-vascular endothelial growth factor (VEGF) therapies in treating macular edema associated with various retinal disorders.

Additionally, in vitreoretinal surgeries, TA enhances the visualization of vitreous structures. When injected into the vitreous cavity, it creates temporary opacification. It enables surgeons to distinguish anatomical structures and pathological changes better, leading to more precise surgical interventions [10]. Beyond enhancing visualization during vitrectomy, TA is critical in reducing postoperative inflammation. Inflammation is a common complication following vitreoretinal procedures, and by mitigating this response, TA helps reduce pain and swelling, accelerating patient recovery times [11]. Moreover, TA effectively suppresses fibrovascular proliferation, a significant factor in developing complications such as epiretinal membranes and tractional retinal detachment. By inhibiting fibroblast activity and collagen deposition, TA contributes to preserving retinal integrity following surgery [11].

Post-traumatic vitreoretinal surgery: Challenges and considerations

Post-traumatic vitreoretinal injuries encompass a range of conditions, including retinal detachments, vitreous hemorrhage, and macular holes. Retinal detachments frequently occur due to tears or breaks in the retina, causing it to separate from the underlying tissue. This detachment can significantly impair visual acuity and, if left untreated, may result in permanent vision loss [12]. Vitreous hemorrhage, characterized by the leakage of blood into the vitreous cavity, can obscure vision and complicate surgical procedures. Traumatic events may also lead to macular holes, which create defects in the macula, severely impacting central vision. These injuries affect visual outcomes and increase the complexity of the surgical techniques required for repair [12]. The impact of trauma on visual acuity is profound, with studies showing that traumatic retinal detachments often result in poorer surgical outcomes compared to non-traumatic causes. Factors such as PVR and extensive scarring can further complicate surgical interventions. As the extent of damage increases, so does the complexity of the surgical techniques required to restore vision. Surgeons must carefully navigate these challenges to optimize visual recovery for their patients [13]. Several surgical techniques are commonly employed to address post-traumatic vitreoretinal injuries. Vitrectomy is one of the most frequently used procedures involving the removal of the vitreous gel to access and repair the retina. This approach is particularly effective for managing both retinal detachments and vitreous hemorrhages. Another surgical method is scleral buckling, which entails placing a silicone band around the eye to indent the sclera and facilitate retinal reattachment. Intraocular tamponades, such as gas or silicone oil, are also used post-surgery to hold the retina in place during healing [5].

Recent advancements in surgical technology have greatly improved these techniques. Introducing smaller gauge instruments has reduced tissue trauma and accelerated patient recovery times. These innovations allow for more precise interventions and contribute to improved outcomes in post-traumatic vitreoretinal surgery [14]. However, significant challenges remain in post-traumatic healing. One major issue is inflammation, which can lead to complications such as PVR, where scar tissue forms on the retina, often necessitating additional surgeries. Fibrosis, or the excessive formation of scar tissue, is another concern, as it can hinder visual recovery and complicate further interventions. Secondary complications, such as recurrent retinal detachment or persistent vitreous hemorrhage, may also occur during the healing process [15]. Addressing these challenges requires a multidisciplinary approach, often including adjunctive therapies, such as corticosteroids particularly TA, to control inflammation and fibrosis. By effectively managing these complications, healthcare providers can improve patient outcomes and enhance the overall success of post-traumatic vitreoretinal surgeries. Ongoing research and innovation in surgical techniques and postoperative care are critical for advancing treatment strategies in this complex field [16]. Table 1 summarizes the key challenges and considerations in post-traumatic vitreoretinal surgery.

**Table 1:** Challenges and considerations in post-traumatic vitreoretinal surgery PVR: Proliferative vitreoretinopathy; TA: Triamcinolone acetonide; IOP: Intraocular pressure

Challenge	Description	Considerations
Complex Anatomy [17]	Post-traumatic eyes often present with disrupted anatomy, including retinal detachment or vitreous hemorrhage.	Careful preoperative imaging is required, and surgical techniques may need to be adapted.
Inflammation and Edema [18]	Trauma induces significant inflammation and edema, complicating both the surgery and recovery.	Anti-inflammatory agents such as TA are often used to control inflammation.
PVR [19]	PVR is a common complication following trauma, leading to membrane formation and retinal retraction.	The use of TA during surgery may reduce the incidence of PVR but close postoperative monitoring is essential.
Vitreous Hemorrhage [20]	Bleeding into the vitreous can obscure the surgeon’s view and complicate intraoperative procedures.	TA can aid in visualization during membrane peeling and vitreous detachment.
Retinal Detachment [12]	Retinal detachment is a common issue in trauma cases, particularly when complicated by tears or proliferative tissue.	Precise membrane peeling and reattachment techniques are required. Postoperative PVR must be monitored.
Choroidal Rupture [21]	Choroidal rupture may result from blunt trauma, leading to severe visual impairment.	Requires careful repair and may necessitate adjunctive treatments such as laser photocoagulation.
Macular Involvement [22]	Macular holes and other macular damage can occur post-trauma, affecting central vision.	TA can assist with membrane peeling in macular hole surgery. Postoperative macular recovery may vary.
IOP Management [23]	Postoperative IOP spikes are common after the use of corticosteroids such as TA.	Regular IOP monitoring and timely intervention with IOP-lowering medication may be necessary.
Scar Tissue Formation [24]	Scar tissue formation can complicate retinal reattachment and reduce visual outcomes.	The use of anti-fibrotic agents and careful surgical techniques can help reduce scarring.
Cataract Formation [25]	Steroid use, including intraoperative TA, can accelerate cataract development in phakic patients.	Consideration for lens removal may be necessary, especially in eyes with traumatic lens damage.
Patient Recovery and Rehabilitation [26]	Postoperative recovery can be prolonged, and visual rehabilitation is often necessary.	Long-term follow-up, including visual therapy, is essential to optimize patient outcomes.

Adjunctive role of triamcinolone acetonide in surgery

TA has become an invaluable adjunct in various surgical procedures, particularly vitreoretinal surgery. Its diverse benefits include enhanced visualization during surgery, effective management of complications, and significant reduction of inflammatory responses. One of the primary uses of TA in vitrectomy is to improve the visualization of vitreous remnants [27]. By providing contrast, TA allows surgeons to more accurately identify and remove these remnants, essential for achieving optimal surgical outcomes. In cases of vitreous hemorrhage, TA can be injected to aid in clearing blood from the vitreous cavity, improving the clarity of the surgical field and enhancing overall procedural efficacy.

Additionally, TA has proven useful during the peeling of epiretinal membranes by reducing tractional forces, making membrane removal easier, and leading to better visual outcomes post-surgery [27]. A key advantage of TA in surgery is its ability to reduce postoperative inflammatory responses. Studies have shown that TA significantly minimizes inflammation and pain following surgery, improving patient comfort and promoting faster recovery [28]. Compared to other agents, patients treated with TA experience markedly lower levels of inflammation. TA has demonstrated superior efficacy in reducing inflammation and facilitating healing among corticosteroids. This is largely due to its potent anti-inflammatory effects, which inhibit leukocyte migration and cytokine release, thereby reducing the body’s inflammatory response [28]. TA also plays a crucial role in preventing PVR, a serious complication that can occur after retinal surgery. TA limits fibroproliferative responses by inhibiting fibroblast proliferation and collagen synthesis, thereby preventing scar tissue formation that could impair visual acuity. Clinical trials have shown that patients treated with TA exhibit lower rates of PVR than those who do not receive this adjunctive therapy, further underscoring TA effectiveness in post-surgical care [19]. Table 2 highlights the adjunctive roles of TA in surgery.

**Table 2:** Adjunctive role of TA in surgery TA: Triamcinolone acetonide; PVR: Proliferative vitreoretinopathy; IOP: Intraocular pressure; ILM: Internal limiting membrane

Surgical Application	Role of TA	Benefits	Considerations
Vitreoretinal Surgery [29]	Enhances visualization of vitreous and membranes	Improved surgical precision; Reduces postoperative inflammation; Decreased incidence of PVR	Risk of increased IOP; Potential cataract formation in phakic patients
Macular Hole Surgery [30]	Facilitates ILM peeling	Aids in the closure of macular holes; Better surgical outcomes in cases of post-traumatic macular damage	Requires precise dosing to avoid complications; Monitoring of IOP needed postoperatively
Epiretinal Membrane Removal [31]	Improves membrane visualization and ease of removal	Clearer view for more accurate membrane removal; Reduced risk of residual tissue	Possibility of retinal toxicity if high doses are used
Management of PVR [19]	Reduces inflammation and visualizes fibrous membranes	Decreases chances of PVR recurrence; Enhances membrane peeling accuracy	Regular IOP monitoring is required due to steroid-induced pressure elevation
Uveitis-related Surgery [32]	Reduces intraocular inflammation and stabilizes the inflammatory environment	Prevents exacerbation of inflammation postoperatively; Helps in controlling intraocular inflammation	Risk of steroid-related side effects, including glaucoma and cataract
Cataract Surgery (Complicated Cases) [33]	Used to prevent or treat postoperative inflammation	Minimizes risk of cystoid macular edema and other inflammatory responses post-surgery	Risk of delayed wound healing; May increase IOP post-surgery
Retinal Detachment Surgery [34]	Used as an adjunct to improve membrane visualization and control inflammation	Helps in identifying and removing the posterior hyaloid; Reduces postoperative inflammation	Requires careful postoperative monitoring for increased IOP and other steroid-related complications
Glaucoma Surgery [35]	Occasionally used for its anti-inflammatory properties in conjunction with other treatments	Helps in controlling postoperative inflammation that can lead to failure of glaucoma filtering surgery	Use with caution, as corticosteroids can also elevate IOP, which could complicate glaucoma
Corneal Transplant Surgery (High-risk Cases) [36]	Reduces graft rejection risk by suppressing inflammation	Lowers the risk of immune rejection in high-risk corneal transplant patients	Prolonged use may lead to steroid-related complications such as glaucoma and delayed corneal wound healing

Current clinical evidence on triamcinolone acetonide in post-traumatic surgery

Several randomized controlled trials (RCTs) have explored TA efficacy in managing post-traumatic conditions within vitreoretinal surgery [37-40]. For instance, the Standard Care vs Corticosteroid for Retinal Vein Occlusion (SCORE) branch retinal vein occlusion (BRVO) trial compared intravitreal TA with standard care for vision loss caused by macular edema secondary to BRVO. The study revealed no statistically significant difference in visual acuity between the groups at 12 months. However, adverse events, such as elevated intraocular pressure (IOP) and cataract development, were more frequent in the higher TA dose group (4 mg) compared to standard care and the lower dose group (1 mg). Similarly, the SCORE central retinal vein occlusion (CRVO) trial assessed TA for CRVO, finding that the 1 and 4 mg doses significantly improved visual outcomes compared to observation alone, with the 1 mg dose exhibiting a superior safety profile [41]. Meta-analyses aggregating data from various studies reinforce the conclusion that intravitreal TA can effectively improve visual outcomes in retinal conditions.

Nevertheless, these analyses emphasize a consistent risk of steroid-related complications, underlining the importance of careful patient selection and monitoring. The evidence suggests that although TA can enhance surgical outcomes, balancing its efficacy with potential risks is critical [42]. Clinical data demonstrate that TA can positively influence visual acuity recovery after vitreoretinal surgery. In trials addressing macular edema, a significant proportion of patients receiving intravitreal TA showed improved visual acuity scores. For example, one study reported that around 29% of participants receiving standard care achieved notable visual gains, with similar rates observed in TA-treated groups. This indicates that TA may be instrumental in accelerating visual function recovery post-surgery [43].

The duration of TA effects varies between studies. While initial improvements in visual acuity are typically observed within weeks, conditions such as cystoid macular edema may recur several months post-treatment. Thus, long-term follow-up is crucial for assessing sustained benefits and monitoring potential complications, providing clinicians with essential information to optimize patient outcomes [44]. One primary concern with TA use is the risk of steroid-induced ocular hypertension. Studies have consistently reported increased IOP following intravitreal injections, particularly at higher doses. In the SCORE trials, elevated IOP was significantly more common in patients receiving the 4 mg dose compared to those on lower doses or standard care, underscoring the importance of close IOP monitoring post-treatment to prevent complications [45]. Cataract formation is another noteworthy adverse effect associated with TA use. In clinical studies, a substantial proportion of phakic eyes treated with TA developed cataracts, highlighting the need for careful preoperative counseling and potential future surgical intervention. Management strategies typically include vigilant IOP monitoring and considering cataract surgery when clinically warranted [46]. Long-term safety profiles of TA remain a focus for ongoing research. While short-term studies indicate effective management of ocular conditions with an acceptable safety profile, more comprehensive long-term data are necessary to fully understand the implications of repeated TA use across diverse patient populations. Continued investigation is essential to establish best practices for TA in post-traumatic vitreoretinal surgery [47].

Future perspectives and emerging trends

Innovations in drug delivery systems are advancing the therapeutic applications of TA, particularly in ophthalmic surgery. One significant development is the creation of sustained-release formulations, which offer prolonged therapeutic effects and more stable drug levels over extended periods [48]. These formulations, developed using matrix systems incorporating hydrophilic and hydrophobic polymers, aim to optimize the TA release profile. This controlled and gradual delivery approach enhances treatment efficacy, minimizes side effects, and improves patient compliance by reducing the frequency of administration [48].

In addition to sustained-release formulations, new injectable forms and implants of TA are emerging as alternatives to traditional routes of administration. These innovations enable more localized therapy, limiting systemic exposure and minimizing adverse effects [49]. For example, implantable devices that release TA over time can offer targeted, long-term treatment for chronic conditions such as post-surgical inflammation or scarring. This localized delivery improves therapeutic outcomes by focusing the effects of the drug on the target area, reducing systemic complications [49].

Another exciting trend is combination therapy wherein TA is used with other treatments such as anti-VEGF agents. This synergistic approach is gaining traction, particularly in managing ocular diseases such as diabetic macular edema and age-related macular degeneration. By simultaneously addressing inflammation and neovascularization, combination therapy offers enhanced efficacy. Evidence suggests that combining TA with anti-VEGF therapies improves visual outcomes, while co-administration with other anti-inflammatory agents can reduce fibrosis and excessive scarring after surgery [50].

As personalized medicine becomes more prevalent, there is an increasing focus on genetic predispositions that influence individual responses to TA therapy. Emerging research indicates that genetic factors are crucial in determining patient outcomes. Pharmacogenomic studies are beginning to identify biomarkers that predict how patients will respond to TA, potentially allowing clinicians to tailor treatment strategies based on genetic profiles [51]. This personalized approach to therapy can optimize efficacy, reduce side effects, and ensure patients receive the most appropriate care for their condition.

The future of TA therapy will likely see greater integration of personalized medicine with innovative drug delivery systems and combination treatments. By customizing therapies based on a patient’s genetic makeup, medical history, and specific disease characteristics, clinicians can significantly improve treatment effectiveness while minimizing risks. This shift toward personalized, targeted therapies has the potential to revolutionize the management of post-traumatic conditions in vitreoretinal surgery, offering enhanced patient outcomes [52]. Table 3 illustrates the future perspectives and emerging trends in the surgical use of TA.

**Table 3:** Future perspectives and emerging trends in the use of TA in surgery TA: Triamcinolone acetonide; IOP: Intraocular pressure; VEGF: Vascular endothelial growth factor; PVR: Proliferative vitreoretinopathy; AI: Artificial intelligence; ENT: Ear, nose, and throat

Emerging Trend	Description	Potential Benefits	Challenges/Considerations
Sustained-Release Drug Delivery Systems [53]	Development of implants or microparticles for controlled release of TA over an extended period.	Prolonged therapeutic effects. Reduced need for repeated injections; Minimized systemic exposure.	Potential for device migration or degradation. Risk of over-suppression of inflammation in prolonged exposure.
Lower-Dose Formulations [54]	Optimizing TA dosage to balance effectiveness with side-effect profile.	Reduced risk of elevated IOP and cataract formation. Safer for prolonged use.	Ensuring sufficient therapeutic impact without compromising efficacy. Tailoring to individual patient needs.
Combination Therapies [55]	Co-administration of TA with other agents such as anti-VEGF, immunomodulators, or antibiotics.	Synergistic effects: Improved treatment outcomes for conditions such as PVR or uveitis.	Complex dosing regimens. Risk of increased side effects from combination therapies.
TA in Minimally Invasive Surgery [56]	TA in smaller doses is used for emerging minimally invasive vitreoretinal and ocular surgeries.	Improved outcomes in less invasive procedures, faster recovery times, and lower complication rates.	Limited data on long-term effects. Needs further clinical trials for validation.
Biodegradable Implants for Localized TA Delivery [57]	Biodegradable implants are designed to release TA directly at the site of inflammation or surgical repair.	Targeted drug delivery, reduced systemic exposure, and enhanced therapeutic effect on localized inflammation.	Development and cost of biodegradable materials. Monitoring of degradation rate and drug release consistency.
Gene Therapy Integration [58]	Exploration of TA in conjunction with gene therapy for conditions such as PVR and retinal degeneration.	Potential for long-term, sustained treatment effects. Reduced need for repeated pharmacological interventions.	High cost and complexity of gene therapy. Ethical and regulatory challenges.
TA in Robotics-Assisted Surgery [59]	Investigating the role of TA in precision-based surgeries using robotics-assisted platforms.	Enhanced surgical precision with improved visualization during membrane peeling and vitreous detachment.	The high initial cost of technology. Training surgeons in using robotics and adjunctive therapies such as TA.
AI in TA Dosing [60]	AI algorithms to optimize TA dosing based on individual patient profiles and surgical needs.	Personalized dosing regimens, reduced risk of side effects, and optimized surgical outcomes.	Development of reliable AI systems for healthcare. Integration with clinical practice.
Expanded Use in Non-Ocular Surgeries [61]	Investigating the role of TA in surgeries beyond ocular procedures, such as orthopedic or ENT surgeries.	Potential anti-inflammatory and healing benefits in various surgical fields. Reduced postoperative complications.	Further research is needed on safety and efficacy in non-ocular surgical applications.

Challenges and limitations in clinical practice

The integration of adjunctive therapies such as TA in post-traumatic vitreoretinal surgery encounters several challenges and limitations that impede widespread adoption and optimal clinical outcomes [62]. One of the key barriers to adopting TA in clinical practice is the cost associated with its use. While TA is relatively affordable, the total financial burden encompasses the cost of the drug and the expenses related to surgical procedures, follow-up care, and the management of potential complications. These cumulative costs can be prohibitive for certain healthcare facilities, particularly those operating under constrained budgets or in resource-limited settings. Furthermore, access to TA may be restricted when patients lack insurance coverage for such treatments or when healthcare systems cannot offer comprehensive care involving adjunctive therapies [63]. Another significant limitation is the variability in surgical techniques among practitioners, leading to inconsistent outcomes when TA is used as an adjunctive treatment. Surgeons may differ in administration, dosing, and timing relative to surgery, substantially affecting the therapy’s efficacy. This inconsistency hampers the development of standardized protocols and guidelines for TA use, making it difficult for clinicians to achieve optimal results across diverse patient populations [64]. The current body of research on the efficacy of TA in vitreoretinal surgery is also limited by the absence of large-scale, multicenter trials. Many existing studies are small and conducted at single centers, raising concerns about the generalizability of their findings. Larger, more comprehensive trials are necessary to confirm the benefits of TA across different demographics and clinical settings, thus providing stronger evidence to support its routine use as an adjunctive therapy [65].

Additionally, there is a significant knowledge gap regarding the long-term outcomes associated with TA use in post-traumatic vitreoretinal surgery. While short-term advantages, such as reduced scarring and improved visual acuity, have been reported, the long-term effects remain poorly understood. Gaining insight into these outcomes is critical for evaluating the overall risk-benefit profile of TA, particularly about potential side effects or complications that may develop after extended use [66]. Table 4 outlines the challenges and limitations associated with the clinical application of TA.

**Table 4:** Challenges and limitations in the clinical practice of TA use IOP: Intraocular pressure; TA: Triamcinolone acetonide; PVR: Proliferative vitreoretinopathy

Challenge/Limitation	Description	Clinical Impact	Considerations for Mitigation
Elevated IOP [67]	TA can lead to transient or prolonged increases in IOP, potentially causing glaucoma.	Risk of optic nerve damage, visual field loss, and long-term complications in susceptible patients.	Regular IOP monitoring post-injection. Use of IOP-lowering medications as needed.
Cataract Formation [68]	Long-term use of corticosteroids such as TA accelerates cataract development, particularly in phakic patients.	Potential need for cataract surgery, especially in patients requiring repeated TA injections.	Careful consideration of TA use in younger, phakic patients. Regular lens evaluation during follow-ups.
Risk of Retinal Toxicity [69]	High doses or prolonged exposure to TA can cause toxicity to the retina.	Retinal damage may impair vision recovery, especially in fragile post-trauma eyes.	Using lower doses, ensuring correct intraocular placement, and monitoring for signs of retinal toxicity.
Limited Duration of Action [70]	Standard TA injections have a limited duration of action, necessitating repeated administration.	Multiple injections increase the risk of cumulative side effects, including IOP spikes and infections.	Development of sustained-release formulations to extend the therapeutic effect while reducing injection frequency.
Steroid-Related Side Effects [71]	Systemic absorption of TA can cause typical steroid-related side effects, such as Cushing's syndrome or hyperglycemia.	It may affect patients with comorbidities such as diabetes, increasing the risk of systemic complications.	Consider alternative agents for high-risk patients. Close monitoring of systemic effects in susceptible individuals.
Postoperative Inflammation Recurrence [72]	While TA helps reduce postoperative inflammation, in some cases, inflammation recurs once the drug’s effect diminishes.	This can lead to delayed recovery, PVR formation, and the need for additional surgeries.	Consider combination therapies or longer-acting TA formulations for prolonged inflammation control.
Infection Risk [73]	Intraocular injections carry the risk of introducing infection, including endophthalmitis.	Severe visual impairment or vision loss if infections are not promptly treated.	Strict aseptic techniques during injection. Close postoperative monitoring for signs of infection.
Patient Compliance [74]	Repeat injections or close follow-up visits for IOP monitoring and inflammation control may be challenging for some patients.	Missed follow-ups can lead to unmanaged side effects or complications, worsening the overall prognosis.	Clear communication with patients about the importance of follow-up care. Simplifying treatment regimens where possible.
Cost and Accessibility [75]	TA injections, especially sustained-release formulations, may be costly and less accessible in low-resource settings.	Limited access to TA therapy may hinder optimal treatment outcomes, particularly in developing regions.	Consider alternative anti-inflammatory therapies in resource-constrained settings. Seek cost-effective solutions.
Contraindications in Glaucoma Patients [76]	TA may not be suitable for patients with pre-existing glaucoma due to its IOP-elevating effects.	Worsening of glaucoma leads to further vision loss and complications.	Alternative anti-inflammatory agents, such as continuous IOP monitoring, should be considered for glaucoma patients.

## Conclusions

In conclusion, TA has proven to be a valuable adjunctive therapy in post-traumatic vitreoretinal surgery, offering significant benefits in enhancing surgical precision, reducing inflammation, and improving postoperative outcomes. Its ability to provide intraoperative clarity by staining the vitreous and epiretinal membranes has made it an indispensable tool for vitreoretinal surgeons, particularly in complex trauma cases. Moreover, TA’s potent anti-inflammatory properties help to mitigate the risk of PVR and other complications that can compromise the success of surgery and long-term visual recovery. While current evidence supports its efficacy, the future of TA in vitreoretinal surgery lies in continued research on its long-term safety profile, innovative drug delivery systems, and potential for combination therapies. As our understanding of post-traumatic ocular healing deepens, TA is likely to remain at the forefront of adjunctive therapies, helping to improve patient outcomes and expand the possibilities of surgical interventions in the management of vitreoretinal trauma.
